# Biocompatible Nanoparticles Based on Amphiphilic Random Polypeptides and Glycopolymers as Drug Delivery Systems

**DOI:** 10.3390/polym14091677

**Published:** 2022-04-20

**Authors:** Natalia Zashikhina, Mariia Levit, Anatoliy Dobrodumov, Sergey Gladnev, Antonina Lavrentieva, Tatiana Tennikova, Evgenia Korzhikova-Vlakh

**Affiliations:** 1 Institute of Macromolecular Compounds, Russian Academy of Sciences, Bolshoy pr. 31, 199004 St. Petersburg, Russia; nzashihina@bk.ru (N.Z.); musia_1@yahoo.com (M.L.); anatoliy.dob@gmail.com (A.D.); 2 Institute of Chemistry, Saint-Petersburg State University, Universitesky pr. 26, 198504 St. Petersburg, Russia; st069020@student.spbu.ru (S.G.); tennikova@mail.ru (T.T.); 3 Institute of Technical Chemistry, Gottfried-Wilhelm-Leibniz University of Hannover, 30167 Hannover, Germany; lavrentieva@iftc.uni-hannover.de

**Keywords:** polypeptides, synthetic glycopolymers, random and block-random copolymers, amphiphilic copolymers, polymer particles, cellular uptake of particles, drug delivery systems

## Abstract

In this research, the development and investigation of novel nanoobjects based on biodegradable random polypeptides and synthetic non-degradable glycopolymer poly(2-deoxy-2-methacrylamido-d-glucose) were proposed as drug delivery systems. Two different approaches have been applied for preparation of such nanomaterials. The first one includes the synthesis of block-random copolymers consisting of polypeptide and glycopolymer and capable of self-assembly into polymer particles. The synthesis of copolymers was performed using sequential reversible addition-fragmentation chain transfer (RAFT) and ring-opening polymerization (ROP) techniques. Amphiphilic poly(2-deoxy-2-methacrylamido-d-glucose)-b-poly(l-lysine-*co*-l-phenylalanine) (PMAG-b-P(Lys-*co*-Phe)) copolymers were then used for preparation of self-assembled nanoparticles. Another approach for the formation of polypeptide-glycopolymer particles was based on the post-modification of preformed polypeptide particles with an oxidized glycopolymer. The conjugation of the polysaccharide on the surface of the particles was achieved by the interaction of the aldehyde groups of the oxidized glycopolymer with the amino groups of the polymer on particle surface, followed by the reduction of the formed Schiff base with sodium borohydride. A comparative study of polymer nanoparticles developed with its cationic analogues based on random P(Lys-*co*-d-Phe), as well as an anionic one—P(Lys-*co*-d-Phe) covered with heparin––was carried out. In vitro antitumor activity of novel paclitaxel-loaded PMAG-*b*-P(Lys-*co*-Phe)-based particles towards A549 (human lung carcinoma) and MCF-7 (human breast adenocarcinoma) cells was comparable to the commercially available Paclitaxel-LANS.

## 1. Introduction

Drug delivery systems are an extremely promising tool for therapeutic approaches such as enhancing *transport* of therapeutics across biological barriers, targeted drug delivery, as well as cancer treatment and gene therapy [1,2,3,4]. The development of delivery systems of antitumor drugs is the leading area of such research. The interest in nanocarriers for preparation of cancer treatments can be related to their ability to increase the solubility and bioavailability of a drug, as well as the local drug concentration in cancer cells [5]. Moreover, the nanoparticles (NPs) can enhance the drug safety and improve drug efficacy, reduce side effects [6,7], prolong the circulation half-life, and maintain the required drug concentration due to controlled drug release. The loading of drugs into the nanocarrier can be achieved via physical encapsulation, chemical conjugation, or a combination of both methods [8,9]. Additionally, electrostatic interactions can also be applied to facilitate encapsulation of drugs [10,11].

The most promising forms of antitumor drug carriers are polymer particles, such as nanospheres, nanocapsules, micelles, liposomes, and polymersomes [12,13,14]. Inorganic gold and iron nanoparticles are used mostly for biovisualization and diagnostics [15,16] but they have significant limitations associated with low loading and poorly controlled drug release [17]. Liposomes, based on natural phospholipids, are the most studied class of nanoparticles, and currently there are a number of commercially available liposomal forms of antitumor drugs (Daune-Home, Lipodox, Onivyde, etc.), which confirm their high efficiency. Despite the advantages of liposomes, one of their significant drawbacks is low stability in a bloodstream. The creation and application of delivery systems based on natural biodegradable polymers such as chitosan [18], dextran [19], alginate [20], gelatin [21], heparin (HEP) [22], and poly-l-lysine (PLys) [23] are described in current literature. Naturally derived polysaccharide and proteins exhibit excellent biocompatibility since they enzymatically degraded into easily metabolized peptides or oligosaccharides in the body, and this degradation rate can be tuned for a desired release profile [24]. However, these polymers often require chemical modification to act as efficient nanocarriers and must be extensively purified to avoid immunogenicity [25].

The application of amphiphilic copolymer makes it possible to control and adjust the physicochemical and biological properties of polymer particles, such as size, morphology, charge, hydrophobicity, stability, biocompatibility, biodegradability, the ability to encapsulate certain substances and to change their characteristics in response to external factors, the presence of chemically reactive groups, which allow surface modification, etc. [26,27,28]. The most widely applied biodegradable amphiphilic polymers include poly(d,l-lactic acid) (PLA), poly(d,l-lactic-*co*-glycolic acid) (PLGA), poly(ε-caprolactone) (PCL), cyclodextrins, and poly(β-amino esters) [25,29].

One of the prospective classes of synthetic biodegradable polymers is poly(amino acids) (or synthetic polypeptides). In contrast to other synthetic polymers, poly(amino acids) have a tendency to form the ordered stable conformations (α-helixes and β-sheets) [30,31]. The diversity of amino acids allows preparation of polypeptides bearing functional groups suitable for further modification with the ligands and allows obtaining systems that are able to change their properties in response to external factors. The variation of amino acids, their sequences and polymerization degree allow adjusting the hydrophilic-lipophilic balance of the polymer and, consequently, obtaining particles with variable structures and morphology. In addition, polypeptides are stable towards hydrolysis, but undergo degradation in vivo by specific enzymes [32]. In spite of intensive development of polypeptide-based nanoparticles, the use of drug delivery systems based on such materials in clinical practice is scarce. The examples currently undergoing clinical trials include anticancer drugs encapsulated in PEG-polypeptide-based carriers: doxorubicin (NK-911), SN-38 (NK012), paclitaxel (NK105), cisplatin (NC-6004), docetaxel (NC-6301), and oxaliplatin (NC-4016) [32]. These systems possess the drawbacks of low drug loading, low in vivo stability, and uncontrolled drug release rate [33], and require further development in order to obtain pharmaceutical products with higher drug loading and controlled degradation and drug release rates. Recently, random poly(lysine-co-phenylalanine)s [P(Lys-co-Phe)] of different compositions were synthesized, and nanoparticles based on this copolymer were characterized as possible delivery systems for different peptide drugs [34,35].

Polypeptides with charged residues in their structure (e.g., glutamic acid and lysine) can behave like polyanions by binding to plasma components. This obstacle negatively affects the behavior of materials based on them such as an aggregation and rapid elimination from the bloodstream [36]. The introduction of non-ionic polar groups is considered as a solution to increase blood circulation lifetime [37]. It can be achieved by the grafting of PEG chains [38] or modification of amino acid functional groups with short ethylene glycol or nonionic monosaccharides [39]. Moreover, the introduction of sugar moieties or sugar-binding groups increase functionality of prepared materials [40]. Approaches such as *N*-carboxyanhydrides (NCA) polymerization using glycopolymer as a macroinitiator [41] and coupling between polypeptide and glycopolymer [42,43,44] are applied in order to obtain polysaccharide-*b*-polypeptide. Moreover, grafted glycopolypeptides can be synthesized by direct polymerization of glycosylated NCA monomers [45,46,47,48] or post-polymerization modification of polypeptides, containing reactive alkene, alkyne, and azide functional groups in the side chain, with saccharide [49,50,51].

Glycopolypeptides play a critical role in biological functions, including molecular recognition and protection from proteases [45]. Such polymers are the promising candidates for the drug delivery system and tissue engineering [32]. For example, the vesicles formed from diblock copolymer hyaluronan-*b*-poly(γ-benzyl-l-glutamate) had high affinity for the surface glycoproteins of living cells. They have been successfully applied as delivery systems of doxorubicin, demonstrating effective growth inhibition of breast tumor in vivo [43]. In turn, glycosylated polypeptides based on poly(d,l-allylglycine) selectively bind to the plant lectin concanavalin A [51]. Thus, synthetic glycopolypeptides may possess specific carbohydrate–protein interactions that can be useful for targeted tumor therapy. In addition, this modification by saccharide residues will ensure the biocompatibility of the nanocarrier [32] as well as increase the time of its circulation in the bloodstream by reducing the uptake by macrophages [52].

In our previous study, to obtain nanomaterials with improved stability, random copolymers based on α-d-amino acids [35] or hybrid block-copolymers, consisting of hydrophilic non-degradable glycopolymer, namely poly(2-deoxy-2-methacrylamido-d-glucose) (PMAG) and polyleucine or polyphenylalanine (PPhe) [53,54], were synthesized and used for preparation of polymer particles. It was found that d-amino acid and PMAG-containing nanoparticles proved to be more stable against enzymatic degradation than those based only on poly(l-amino acids). Moreover, PMAG-based polymer particles did not induce cell death in a wide range of concentrations and possessed glutathione-mediated particles’ disruption that could vary depending on hydrophilic block length [54].

In order to combine the positive features of synthetic glycopolymers and random polypeptides, we proposed the development of amphiphilic block-random copolymers, namely poly(2-deoxy-2-methacrylamido-d-glucose)-*b*-poly(l-lysine-*co*-l-phenylalanine) [PMAG-*b*-P(Lys-*co*-Phe)] and their characterization as drug delivery systems. Replacing a hydrophobic polypeptide (PPhe) in the hybrid copolymer by a random copolymer [P(Lys-co-Phe)] building block allows the introduction of functional charged groups (NH_2_-groups) in the PMAG-based particle structure. Such copolymer functionalization provides pH-sensitivity and additional ability to polymer modification by molecules of interests, e.g., by vectors for targeted delivery that is important for creation of the controlled drug delivery and release. In turn, PMAG as a hydrophilic block contributes to partial shielding of the charge of the random polypeptide core, which increases the stability of particles in the presence of proteins and enzymes, thereby increasing their biocompatibility.

Synthesis of PMAG-*b*-P(Lys-*co*-Phe) with different length of blocks was performed using consistently two polymerization techniques: reversible addition-fragmentation chain transfer (RAFT) for PMAG synthesis, and ring-opening polymerization (ROP) of a mixture of α-amino acid *N*-carboxyanhydrides (NCAs). In this case, glycopolymer and random polypeptide blocks were linked with redox-responsive disulfide bond, which can be cleaved at intracellular glutathione concentration, leading the collapse of the particles and release loaded drug in reducing environment of the cell. Another approach proposed for preparation of glycopolymer–polypeptide particles is based on the covalent covering of amphiphilic polypeptide core with hydrophilic non-degradable PMAG. The conjugation includes the interaction of amino groups of the particle surface and the aldehyde groups of the oxidized PMAG, followed by the reduction of the formed Schiff base with sodium borohydride with the formation of more stable single bonds.

The synthesized copolymers demonstrated the ability to self-assemble into nanoparticles whose hydrodynamic diameter, polydispersity index, and ζ-potential were determined using dynamic and electrophoretic light scattering. The nanoparticles were studied in terms of their cytotoxicity, kinetics of penetration into cancer cells, and the uptake by macrophages, as well as biological activity of the encapsulated forms of paclitaxel to evaluate and compare their perspectives as delivery systems. The properties of the PMAG-*b*-P(Lys-*co*-Phe) nanoparticles were compared to those determined for the polypeptide precursor, namely random P(Lys-*co*-d-Phe), and this one covered with a natural polysaccharide.

## 2. Materials and Methods

### 2.1. Materials

Triphosgene (98%), α-pinene (98%), hexylamine (HexNH_2_, 99%), *N*_6_-Carbobenzyloxy-l-lysine (Lys(Z), ≥99%), L-phenylalanine (Phe, ≥98%), d-phenylalanine (d-Phe, ≥98%), 2-aminoethanethiol hydrochloride (AETL, cystamine hydrochloride, ≥98%), sodium borohydride (≥96%), 4-Cyano-4-(phenylcarbonothioylthio)pentanoic acid (CTA), trifluoroacetic acid (TFA, 99%), hydrogen bromide solution (33 wt% in acetic acid), *N*-hydroxysuccinimide (NHS, 98%), *N*-(3-Dimethylaminopropyl)-*N*′-ethylcarbodiimide hydrochloride (EDC, ≥99%), sodium (meta)periodate, papain were purchased from Sigma-Aldrich (Schnelldorf, Germany) and used as received. Heparin (HEP, 8000-25,000) was a product of AppliChem (Darmstadt, Germany). 2,2′-Azobisisobutyronitrile (AIBN, Acros Organics, Geel, Belgium, 98%) was purified by recrystallization from ethanol and dried under vacuum.

Solvents: *N,N*-dimethylformamide (DMF), 1,4-dioxane, ethyl acetate, methanol, ethanol, chloroform, n-hexane, triethylamine (TEA), and other ones used in this work were purchased from Vecton Ltd. (St. Petersburg, Russia) and distilled before use.

Membranes for dialysis with molecular weight cut-off (MWCO) 1000, 3500, 6000–8000, and 12,000–14,000 were purchased from Orange Scientific (OrDial D-Clean regenerated cellulose dialysis tubing, Belgium). Amicon^®^ Ultra 0.5 mL Centrifugal Filters (regenerated cellulose 3000, 10,000, 30,000 Devices) were used for ultrafiltration (Merck, Darmstadt, Germany).

Human embryonic kidney cells (HEK 293), human lung carcinoma cells (A549), mouse macrophages (J774A.1), and human bronchial epithelial cells (BEAS-2B) were purchased from CLS Cell Lines Service GmbH (Eppelheim, Germany). HEK 293, A549, and J774A.1 were cultivated in Dulbecco’s Modified Eagle’s Medium (DMEM, Merck, Darmstadt, Germany) and BEAS-2B was grown in LHC-9 (Thermo Fisher Scientific, Dreieich, Germany) medium, both media were supplemented with 10% (*v*/*v*) fetal calf serum (FCS) (Biochrom, Berlin, Germany), and 1% (*v*/*v*) penicillin/streptomycin (P/S) (Biochrom, Berlin, Germany). MCF-7 (human breast adenocarcinoma) cells were purchased from German Collection of Microorganisms and Cell Culture (Braunschweig, Germany) and grown in Modified Eagle’s Medium (Merck, Germany) containing 10% (*v*/*v*) FCS, 1% L-glutamine, 1% sodium pyruvate, 50 U/mL penicillin, and 50 µg/mL streptomycin, 0.1% nonessential amino acids (NEA) (Biochrom, Berlin, Germany), and 1 µM insulin (Merck, Darmstadt, Germany). CellTiter-Blue cell viability assay reagent was purchased from Promega (Madison, WI, USA). Cyanine3 NHS ester (Cy_3_-NHS, 95%) and Cyanine5 amine (Cy_5_-NH_2_, 95%) were products of Lumiprobe (Moscow, Russia).

### 2.2. Synthesis and Characterization of (Co)Polymers

Poly(2-deoxy-2-methacrylamido-d-glucose) (PMAG) was obtained via reversible addition-fragmentation chain transfer (RAFT) as described in our previous studies [53]. For the synthesis of oxidized PMAG (ox-PMAG), the following procedure was applied. Dithiobenzoate end-group of the polymer was removed by reduction with NaBH_4_, followed by dimerization of the polymer in the presence of iodine. Briefly, to the solution of 0.2 g of PMAG in 5 mL DMF purged with argon, NaBH_4_ (100 eq relatively to the chain end-groups) was added under stirring and inert atmosphere. When the pink color of the solution was disappeared, the mixture was stirred for additional 1 h. Thiol groups of polymers were then oxidized with the formation of disulfide bond using the 1 M solution of iodine in DMF. The oxidizing agent was added until a permanent yellow color appeared. The unreacted iodine was reduced with ascorbic acid. The product was purified by dialysis (MWCO = 12,000) against deionized water. PMAG was then oxidized with NaIO_4_ to form dialdehyde derivatives (ox-PMAG) as described elsewhere [55]. The amount of aldehyde groups was varied by the setting the initial molar ratio of NaIO_4_/MAG units from 0.1 to 0.5. Briefly, 0.1 g of polymer was dissolved in 16 mL of distilled water and cooled to 4 °C, and the sodium periodate was added with vigorous stirring. The mixture was kept for 24 h in the dark at 4 °C, then the product (ox-PMAG) was dialyzed against distilled water and lyophilized. Synthesis of PMAG-*b*-P(Lys-*co*-Phe) was fulfilled by ring-opening polymerization (ROP) of a mixture of corresponding α-amino acid *N*-carboxyanhydrides (NCAs) using PMAG-NH_2_ (PMAG bearing terminal NH_2_-group) as a macroinitiator (macroI). Synthesis of PMAG-NH_2_ was carried out according to the procedure published elsewhere [54]. For copolymerization, l-Lys(Z) NCA (M_1_) and l-Phe NCA (M_2_) were preliminarily dissolved together in DMF (6 wt%). After that, solution of macroinitiator PMAG-NH_2_, in DMF was added with stirring in the monomer solution, to achieve the predetermined ratio [M_1_ + M_2_]/[macroI]. All the solutions were preliminary purged with argon for 30 min. Then the polymerization mixture was shaken at 25 °C for 72 h. To remove unreacted monomers and macroinitiator, the resulting copolymers were dialyzed using the membrane with MWCO 6000–8000 against water. After purification, the solution was freeze-dried. Depending on composition the polymer yield ranged from 28 to 73%.

The composition of copolymers was calculated from ^1^H NMR spectra. The spectra were recorded at 25 °C in DMSO-d_6_ using a Bruker AC-400 NMR spectrometer (400 MHz) (Karlsruhe, Germany). For the calibration of the chemical shift scale of the NMR spectra, the solvent peak at 2.52 ppm was used.

The ratio of relative integral areas of the aromatic protons of the carboxybenzyl group of Lys(Z) (7.32 ppm) and the aromatic protons of Phe (7.2 ppm) were used to calculate the ratio of monomer amino acid units to each other in the copolymer:

(1) $$\left[\mathrm{Lys}\left(Z\right)\right]/\left[\mathrm{Phe}\right]=\left(\frac{I{\left(\mathrm{Lys}\left(Z\right)\right)}_{7.32\mathrm{ppm}}}{I{\left(\mathrm{Phe}\right)}_{7.2\mathrm{ppm}}}\right)$$

where I(Lys(Z))_7.32ppm_ and I(Phe)_7.2ppm_ are relative integral areas of 5 aromatic protons of Phe and Z-groups of the polymer at 7.2 and 7.32 ppm, respectively.

The ratio of Lys(Z) to MAG units was calculated from the ratio of the signals of aromatic protons Lys(Z) (7.32 ppm) and the total signal intensity of methyl and methylene protons PMAG and 6 protons Lys(Z) at 0.7–2.0 ppm:

(2) $$\left[\mathrm{Lys}\left(Z\right)\right]/\left[\mathrm{MAG}\right]=\left(\frac{I{\left(\mathrm{Lys}\left(Z\right)\right)}_{7.32\mathrm{ppm}}}{I{\left(\mathrm{Lys}\left(Z\right)\right)}_{0.7-2.0\mathrm{ppm}}+I{\left(\mathrm{PMAG}\right)}_{0.7-2.0\mathrm{ppm}}-\frac{I{\left(\mathrm{Lys}\left(Z\right)\right)}_{7.32\mathrm{ppm}}}{5}\times 6}\right)$$

where I(Lys(Z))_7.32ppm_, I(Lys(Z)_0.7–2.0ppm and_ I(PMAG)_0.7–2.0ppm_ are relative integral areas of 5 aromatic protons of Lys(Z) at 7.32 ppm, and 6 aliphatic proton of Lys(Z) and methyl and methylene protons PMAG at 0.7–2.0 ppm.

Synthesis and characterization of random copolymers poly(L-lysine-*co*-d-phenylalanine) (P(Lys-*co*-d-Phe)) applied for comparison were performed using a previously published protocol [35].

The characteristics of (co)polymers (molecular weights *M_n_* and *M_w_* and dispersity *Ð*) were determined by size exclusion chromatography (SEC) using Shimadzu LC-20 Prominence system equipped with refractometric RID 10-A detector (Kyoto, Japan). The analysis was carried out at 60 °C using 0.1 M LiBr in DMF as eluent (0.3 mL/min) and Styragel Column, HMW6E (7.8 mm × 300 mm, 15–20 µm bead size, Waters, Milford, MS, USA). Calculations were made using GPC LC Solutions software (Shimadzu, Kyoto, Japan) and preliminary built calibration curve for poly(methyl methacrylate) standards with M*_w_* 17,000–250,000 (*Ð* ≤ 1.14).

The deprotection of NH_2_-groups of the synthetized copolymers was performed using HBr/TFA mixture. 2 mL of TFA was added to 100 mg of the copolymer and stirred for 60 min while cooling with an ice bath, after which 1 mL of a 33% solution of HBr in CH_3_COOH was added. The reaction was carried out with stirring for 6 h. Then, 10 mL of DMF was added to the reaction mixture and the resulting solution was transferred into a dialysis bag (MWCO 1000) and dialyzed against water for 48 h.

The ratio of amino acids in deprotected copolymers was estimated using HPLC analysis after acidic hydrolysis of copolymer. For hydrolysis, a 6 M HCl solution containing 0.0001% phenol was prepared. A weighed portion of the copolymer was placed into the ampoule and a solution for hydrolysis was added in the ratio of 1 mL/0.5 mg of the copolymer. Then, the ampoule was sealed, and the mixture was kept for 4 d at a temperature of 120 °C.

After that, the solvent was evaporated, the residue was dissolved in water, and evaporated again. The procedure was repeated several times until a neutral pH of the solution was obtained. The resulting solution was analyzed using ion-exchange HPLC with refractometric detection.

The hydrolysates were analyzed using 4.6 × 125 mm SHODEX IC YS-50 column (Phenomenex, Torrance, CA, USA) packed with 5 µm particles. The isocratic elution mode was applied using 6 mM H_3_PO_4_ as eluent. The mobile phase flow rate was equal to 1.0 mL/min and analysis time—15 min. Quantitative determination of the concentration of amino acids was carried out using the preliminary built calibration plots in the range of amino acid concentrations of 0.005–0.5 μg/mL. The retention times of Phe and Lys were 5.2 and 10.7 min, respectively.

### 2.3. Preparation and Characterization of Nanoparticles

Polymers’ self-assembly was achieved during gradient phase inversion (dialysis) of polymer solution (see Section 2.2. Synthesis and Characterization of (Co)Polymers). Then nanoparticles (NPs) were freeze-dried and stored at 4 °C. Colloids of NPs were prepared by dispersing the dried NPs for 60 s under sonication in 0.01 M PBS (pH 7.4). The colloids were characterized by dynamic light scattering (DLS), measuring the hydrodynamic diameter (*D_H_*), polydispersity index (PDI), and the ζ-potential of NPs using a ZetasizerNano-ZS (Malvern, UK) equipped with a He–Ne laser at 633 nm at a scattering angle of 173° and 25 °C. The size of NPs in dry state was studied by scanning transmission electron microscopy (STEM) using a UHR FE-SEM SU8030 (Hitachi, Tokyo, Japan). Before analysis, a few drops of a colloid in PBS (0.01 M, pH 7.4) were placed onto a nickel grid covered by carbon and dried. Then, the grid was stained with 1% (*w*/*v*) uranyl acetate solution for 30–60 s and was used for the experiments after 24 h.

To study the stability of NPs in protein-containing medium, the colloids of polymer nanoparticles were prepared by dispersing 1 mg of the particles in 1 mL of PBS (0.01 M, pH 7.4) under sonication for 60 s. The solution was then tenfold diluted with DMEM and incubated for 24 h at 37 °C. The hydrodynamic diameter of NPs was determined by DLS.

Stability of NPs to biodegradation was studied in model medium representing 0.01 M PBS, pH 7.4, containing 0.5 mg/mL of papain. The mixture was incubated at 37 °C for 35 days. At predetermined time intervals, the hydrodynamic diameter of NPs was measured by DLS.

### 2.4. Particle Surface Modification

Coating of particles with heparin was carried out as follows. To 1 mL of a heparin solution (1 mg/mL) in water, 5 mL of the particle suspension (0.1 mg/mL) was added with vigorous stirring. Unbound heparin was removed via ultrafiltration with the use of tubes supplied with membranes of 50,000 MWCO.

The covalent modification of the surface of particle based on a random copolymer of poly(L-lysine-*co*-dl-phenylalanine) was carried out using oxidized poly(2-deoxy-2-methacrylamido-d-glucose) (ox-PMAG). Covalent coupling reaction was proceeded in sodium borate buffer, pH 8.5, at room temperature with stirring. To 1 mL of the solution of PMAG (sodium borate buffer, pH 8.5), 1 mL of particles was added with vigorous stirring, and the reaction was allowed to proceed for 2 h. While the initial PMAG concentration was varied from 0.02 to 0.6 mg/mL, the particle concentration was fixed at 0.2 mg/mL. After that, the formed particles were treated with sodium borohydride (10-fold excess relatively to aldehyde groups) for 20 h. A 10% solution of acetic acid in water was added to the resulting mixture for additional 2 h (in 2-fold relatively to NaBH_4_). The particles were purified via dialysis against deionized water using dialysis membrane with 12,000–14,000 MWCO to remove low-molecular impurities and unbounded PMAG.

The fluorescently labeled nanoparticles were prepared with the aim of their further visualization. Preparation of fluorescently labeled particles was carried out in different ways for different types of particles depending on the method of their formation. In particular, poly(lactic acid)-based particles (PLA) were obtained by nanoprecipitation; therefore, their surface was modified after particle formation. In turn, the particles based on polypeptides can be obtained in the process of phase inversion (dialysis) due to the self-assembly of the polymer. Thus, the polypeptide was labeled in organic solvent, followed by the particles’ formation by the phase inversion method.

The covalent modification of the particles’ surface was carried out using Cy_3_-NHS or Cy_5_-NH_2_ dyes. For preparation of fluorescently labeled polypeptide-based NPs, 2 mg of the copolymer were dissolved in 1 mL of DMSO and 50 μL of a Cy_3_-NHS solution in DMSO (1 mg/mL) were added. The reaction mixture was stirred at room temperature for 24 h in the dark to prevent a fluorescence quenching. The excess of the dye was removed by dialysis (MWCO 1000) against water for 48 h in the dark.

For modification of NPs with Cy_5_-NH_2_, the Schiff base reaction or the activated ester technique were used. Carboxylic groups of PLA-based NPs were preliminary activated with N-hydroxysuccinimide (NHS) to form activated NHS esters as described elsewhere [56]; or oxidation of glucopyranose ring of poly(2-deoxy-2-methacrylamido-d-glucose) with NaIO_4_ to form reactive aldehyde groups were applied as previously described [57]. Then, 60 μL of a Cy_5_-NH_2_ solution in DMSO (0.1 mg/mL) were added to 2 mL of the NPs colloid (1 mg/mL). The reaction mixture was stirred at room temperature for 24 h in the dark. Unconjugated dye was removed by dialysis (MWCO 1000) against water for 48 h in the dark.

### 2.5. Cell Culture Experiments

HEK 293 (human embryonic kidney cells) and BEAS-2B (human bronchial epithelial cells) were used to determine cytotoxicity of NPs. MCF-7 (human breast adenocarcinoma cells) and A549 (human lung carcinoma cells) were used as an in vitro model to determine the effectiveness of encapsulated drug formulations. J774A.1 (mouse macrophages) and A549 served to study the kinetics of particle cellular uptake. All cells were cultivated in a humidified environment at 37 °C and 5% CO_2_. The medium was changed three times per week and the cells were subcultivated before reaching confluence using trypsin for adherent cells or cell scraper for macrophages J774A.1.

#### 2.5.1. Cytotoxicity

The cell viability was evaluated after 24 or 72 h coincubation of NPs with cells using a previously published protocol for CTB Assay [54]. Concentration-dependent normalized cell viability data obtained from CTB assay were fitted by using non-linear curve fitting/growth/sigmoidal/dose-response fitting functions (OriginPro 8.6). Half-maximal inhibition concentrations (IC_50_) were calculated from the fitted dose-response curves.

#### 2.5.2. Cellular Uptake Study

A qualitative study of the penetration of nanoparticles into cells was performed using fluorescence microscopy. 4 × 10^4^ cells in 200 μL of DMEM were seeded on glass chamber slides (LabTec-II with CC2 treatment) and cultured for 24 h. The medium was then changed with a fresh one, containing fluorescently labeled particles at a concentration of 60 µg/mL. Cells were incubated for 4 h at 37 °C. Thereafter the medium was removed, and cells were washed three times with warm 0.01 M PBS, pH 7.4, and detected under fluorescence microscope (Olympus IX50) equipped with an Olympus camera (SC30, Tokyo, Japan) and Cell Sens Standard software, using excitation, and barrier filters were BP 530–550 and BA 590, respectively.

For quantitative determination of particles’ accumulation in the cells, the method of flow cytometry was used. The 3 × 10^6^ cells in DMEM medium was cultivated in 2 mL medium in 6-well plates for 24 h in a humidified environment at 37 °C and 5% CO_2_ until a 70–80% confluence was reached. Then the medium was removed and replaced with a fresh one containing different concentrations of labeled NPs (0, 5, 10, 15, 25, 50 µg/mL). The exposure was carried out for 24 h in an incubator at 37 °C and 5% CO_2_. In the second approach, the exposure time was varied (0–24 h) with a constant NPs concentration of 25 µg/mL. After the exposure time, the cells were prepared for analysis.

After cultivation and exposure to NPs, cells were washed three times with PBS, detached by cell lifter or using 500 µL trypsin and centrifuged at 300 rpm for 5 min. Finally, they were resuspended in 500 µL PBS and fluorescence signals were measured via flow cytometry. The samples were analyzed by a BD Accuri C6 with a 488 nm Argon-ion laser. Cy_3_ and Cy_5_ fluorescence was collected by a 585/40 band-pass filter. At least 30,000 events per sample were analyzed. Only viable cells were taken for the analysis.

To compare the cellular uptake of particles with different fluorescence intensity, a parameter “accumulation” was introduced as a reference, which was defined as the cell fluorescence intensity I_fl cell_ divided by the fluorescence intensity of the particles (I_fl NPs_). The values of accumulation for each point were calculated as a percentage of value obtained for the nanoparticles based on Cy_3_-labeled P(Lys-*co*-d-Phe) (sample #2.1) at the concentration of NPs of 25 μg/mL after 24 h of incubation or Cy_5_-labeled PLA at the concentration of NPs of 50 μg/mL after 6 h of incubation.

## 3. Results and Discussion

### 3.1. Synthesis and Functionalization of (Co)Polymers

Recently, we have reported the synthesis of biodegradable poly(l-lysine-*co*-d-phenylalanine) (P(Lys-*co*-d-Phe)), which was characterized with prolonged stability to enzymatic cleavage due to the presence of d-amino acid [35]. That cationic polymer formed the narrowly sized nanoparticles prospective as drug delivery systems. The main drawbacks of P(Lys-*co*-d-Phe) NPs were their aggregation in the culture medium and cytotoxicity at concentrations higher than 32 µg/mL. As it has been shown, the mentioned obstacles can be overcome by the covering of P(Lys-*co*-d-Phe) NPs with heparin [34,35]. Thus, the application of hydrophilic negatively charged or neutral glycopolymer can shield the positive charge of P(Lys-*co*-Phe) NPs and reduce the cytotoxicity and a rate of uptake by macrophages. In this regard, the synthetic neutral glycopolymer PMAG covalently conjugated with P(Lys-*co*-Phe) as a hydrophilic block or shell can provide the abovementioned properties as well as improve their stability to enzymatic cleavage and guarantee the stable linkage with P(Lys-*co*-Phe). In turn, an introduction of the disulfide bond between PMAG and P(Lys-*co*-Phe) blocks could provide the sensitivity of such systems to glutathione-mediated disruption of the NPs inside a cell.

In this study, we have continued the development polypeptide-glycopolymer nanomaterials using two other approaches. The first one includes the synthesis of block-random copolymers consisting of polypeptide and glycopolymer capable of self-assembly into polymer particles. The second one is based on the post-modification of preformed polypeptide particles with an oxidized glycopolymer.

**Figure 1:**
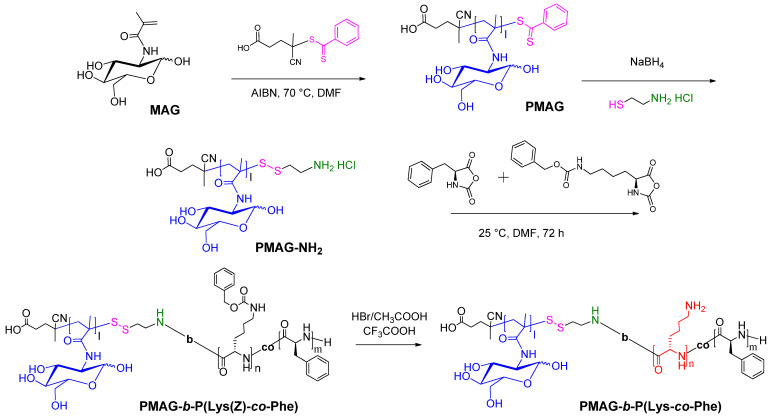
Scheme of synthesis of amphiphilic PMAG-*b*-P(Lys-*co*-Phe).

The synthesis of block-random PMAG-*b*-P(Lys-*co*-Phe) was carried out by a combination of the sequential reversible addition-fragmentation chain transfer (RAFT) and ring-opening polymerization (ROP) techniques [53,54]. The scheme of synthesis for block-random PMAG-*b*-P(Lys-*co*-Phe) is shown in Figure 1. At the first step, the homopolymer PMAG was synthesized via RAFT polymerization using 4-cyanopentanoic acid-4-dithiobenzoate (CTA) as chain transfer agent. At the second step, primary amino group, suitable for ROP polymerization was introduced into PMAG structure. For this, its dithiobenzoate terminal-group was reduced to a thiol followed by oxidation with cystamine hydrochloride. Finally, such homopolymer bearing terminal amino group (PMAG-NH_2_) was used as a macroinitiator for the ring-opening polymerization of the mixture of l-Lys(Z) (M_1_) and l-Phe (M_2_) *N*-carboxyanhydrides to obtain block-random copolymers. After deprotection of Z-protective groups, the desired hybrid amphiphilic copolymers were obtained.

In order to establish the influence of the structure of the copolymer (the length of the neutral hydrophilic block, the composition and length of polypeptide block), the copolymers of various compositions were synthesized. The synthesis of PMAG-NH_2_ was carried using a previously developed protocol [54]. Two samples of PMAG-NH_2_ differed by their molecular weight characteristics were used as the macroinitiators: the first one contained 22 monomer units (*M_n_* = 10,700, *Ð* = 1.09), the second one—80 units (*M_n_* = 25,500, *Ð* = 1.12).

**Figure 2:**
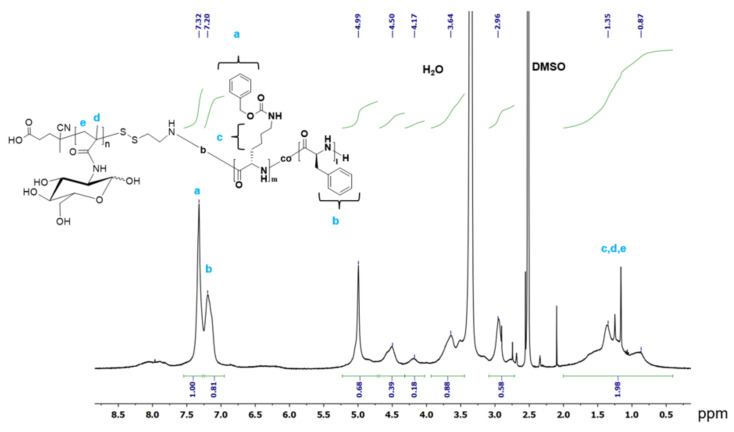
^1^H NMR spectrum of PMAG-*b*-P(Lys(Z)-*co*-Phe) (sample #1.2).

The polymerization conditions and characteristics of obtained protected and deprotected PMAG-*b*-P(Lys-*co*-Phe) of different composition are summarized in Table 1. The molecular weight characteristics of Z-protected copolymers were determined by size exclusion chromatography (SEC). In all cases, the dispersity (*Ð*) of copolymers does not exceed 1.5. The compositions of PMAG-*b*-P(Lys-*co*-Phe) samples were calculated using ^1^H NMR spectra of Z-protected copolymers (Figure 2) as described in the Experimental part. Moreover, the molar ratio of amino acids in the deprotected polymer was established by quantitative amino acid HPLC analysis after total acidic hydrolysis of copolymer. The results obtained by ^1^H NMR spectroscopy and HPLC analysis seem to be in good agreement (Table 1). The ratio of amino acids in the polymer is close to the molar ratio of monomers in the polymerization mixture; however, in all cases, there was a slight deviation towards an increase in Lys units relative to Phe, which is apparently associated with a higher activity of NCA Lys(Z) in the copolymerization reaction. The length of the P(Lys-*co*-Phe) polypeptide fragment in the final block copolymer was comparable to [*M*_1_ + *M*_2_]/[macroI] and decreased with increasing [NCA Phe]/[NCA Lys(Z)] ratio.

**Figure 3:**
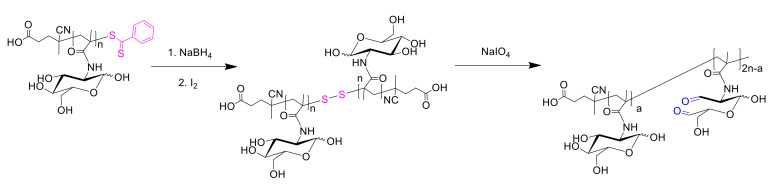
Scheme of synthesis of oxidized PMAG.

The synthesis of oxidized glycopolymer was carried out using a sample of PMAG containing 22 monomer units (*M_n_* = 10,700, *Ð* = 1.09). For the preparation of functionalized PMAG, its dithiobenzoate end-group was removed by reduction with NaBH_4_, followed by dimerization of the polymer in the presence of iodine (Figure 3). Dialdehyde derivatives (ox-PMAG) were then obtained via reaction with IO_4_^−^ ion as described elsewhere [55]. The oxidation was performed at different molar ratios of NaIO_4_/MAG units to vary of aldehyde groups content.

### 3.2. Preparation and Characterization of Polymer Nanoparticles

As it was mentioned above, the introduction of the glycopolymer with neutral saccharide units into the particle structure is needed to partially decrease the surface charge caused by the presence of charged α-amino acids (lysine), which help to enhance the stability of particles with respect to the uptake by macrophages and increase their blood circulation time. Furthermore, it is expected that the introduction of d-amino acids or non-degradable polyvinylsaccharides into the biodegradable polypeptide structure will reduce the enzymatic degradation rate of polypeptide chains, which, as a result, will contribute to the prolonged action of drug encapsulated.

**Figure 4:**
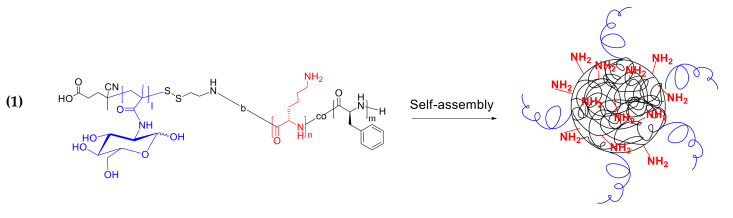

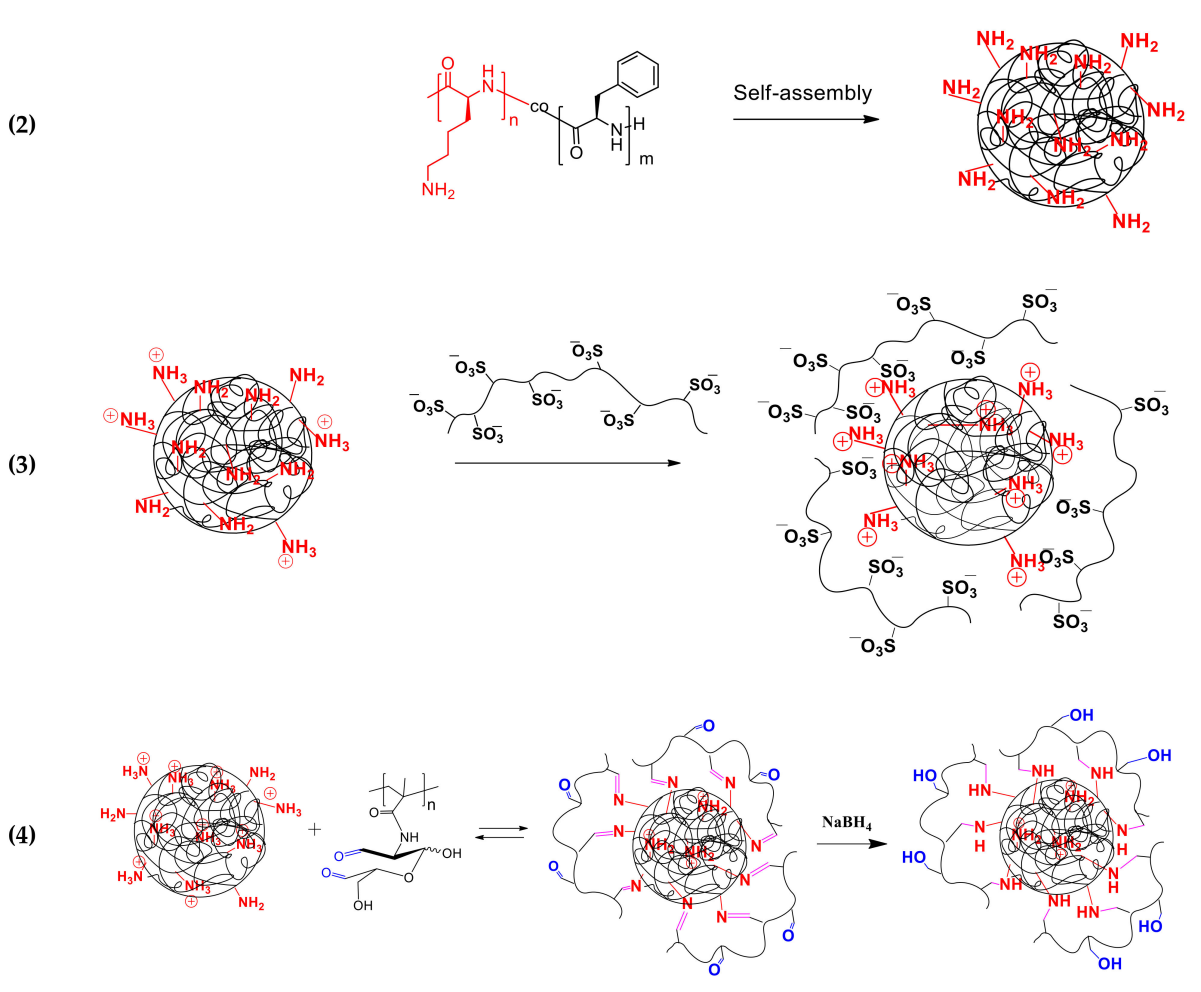
Schematic representation of: (**1**) PMAG-*b*-P(Lys-*co*-Phe) self-assembly; (**2**) P(Lys-*co*-Phe) self-assembly; (**3**) covering of P(Lys-*co*-d-Phe)-based NPs with heparin; (**4**) covalent modification of P(Lys-*co*-dl-Phe)-based NPs with ox-PMAG.

Two methods for obtaining particles, consisting of a glycopolymer hydrophilic non-degradable shell (PMAG) covalently bonded to an amphiphilic polypeptide core, were developed. The NPs based on amphiphilic PMAG-*b*-P(Lys-*co*-Phe) were prepared due to the copolymer self-assembly under the gradient phase inversion conditions (dialysis). To investigate the main patterns between composition of copolymers and properties of the NPs self-assembled from them, the characteristics of developed PMAG-*b*-P(Lys-*co*-Phe) NPs were compared to the precursor P(Lys-*co*-d-Phe) NPs and the same NPs electrostatically covered with heparin (HEP). Schematic representation of the NPs based on PMAG-*b*-P(Lys-*co*-Phe) and P(Lys-*co*-d-Phe) as well as the scheme of P(Lys-*co*-d-Phe) covering with HEP are given in Figure 4.

As well as random polypeptides, the obtained ternary amphiphilic copolymers were able to self-assemble in the aqueous media. In the case of PMAG-*b*-P(Lys-*co*-Phe), the self-assembled nanoparticles consist of a hydrophilic-hydrophobic core formed by random polypeptide due to the hydrophobic interaction of Phe units and a hydrophilic shell whose properties are determined by both the charged PLys fragment and the neutral PMAG block, which partially cover the charged layer from interaction with the external medium.

The morphology of polymer particles was investigated by transmission electron microscopy (TEM) (Figure S1 of Supplementary Materials). All tested nanoparticles had a spherical shape. However, in contrast to random polypeptide-based particles, the ones based on hybrid block-random copolymers had a looser structure.

The hydrodynamic diameter (*D_H_*), polydispersity index (PDI), ζ-potential of nanoparticles obtained were investigated by dynamic and electrophoretic light-scattering (Table 2). As seen from the presented values of *D_H_*, it is obvious that hydrodynamic diameter depended on both the amount of positively charged Lys and the presence glycopolymer. In first case, the enrichment of a polypeptide with lysine contributed to the increase of hydrodynamic diameter due to the repulsion of the same charged polymer fragments. This tendency was observed for both P(Lys-*co*-d-Phe) and PMAG-*b*-P(Lys-*co*-Phe) NPs. An introduction of the PMAG block into copolymer structure provided self-assembling into NPs of the larger *D_H_*: compare sample #2.1 for P(Lys-*co*-d-Phe) and sample #1.2 for PMAG-*b*-P(Lys-*co*-Phe) having close to each other polypeptide composition (Table 2). In turn, an increase in the PMAG length, while polypeptide block length and composition were approximately equal, also led to increase in *D_H_* (see samples #1.2 and #1.5 for PMAG-*b*-P(Lys-*co*-Phe), Table 2). At the same time, an increase in the degradable polypeptide block length, while the amino acid ratio is approximately equal, caused only a slight increase in the hydrodynamic diameter, namely: the particles based on samples #1.2 and #1.4 of PMAG-*b*-P(Lys-*co*-Phe) with approximately equal molar ratio of Phe/Lys in their structure had almost the same *D_H_*. Probably in this case, the uncharged PMAG block, which forms the external hydrophilic shell, to a lesser extent than the Phe/Lys ratio, affected the particle self-assembling. Finally, when the content of the hydrophobic fragment is too high, and sufficient stabilization of core by hydrophilic corona cannot be achieved, the *D_H_* of the particles also increased due to particle aggregation. In particular, *D_H_* and PDI values for the PMAG-*b*-P(Lys-*co*-Phe) NPs based on sample #1.3 molar ratio of Phe/Lys equal to 1.5) almost doubled compared to sample #1.2 (molar ratio of Phe/Lys equal to 1) (Table 2).

Compared to the introduction of PMAG into the copolymer with amphiphilic polypeptide, the coating of P(Lys-*co*-d-Phe) NPs with heparin (P(Lys*-co*-d-Phe)-HEP) also followed by an increase in their hydrodynamic diameter. The coating of NPs with heparin was confirmed by changing the sign of ζ-potential from positive to negative values (Table 2).

**Figure 5:**
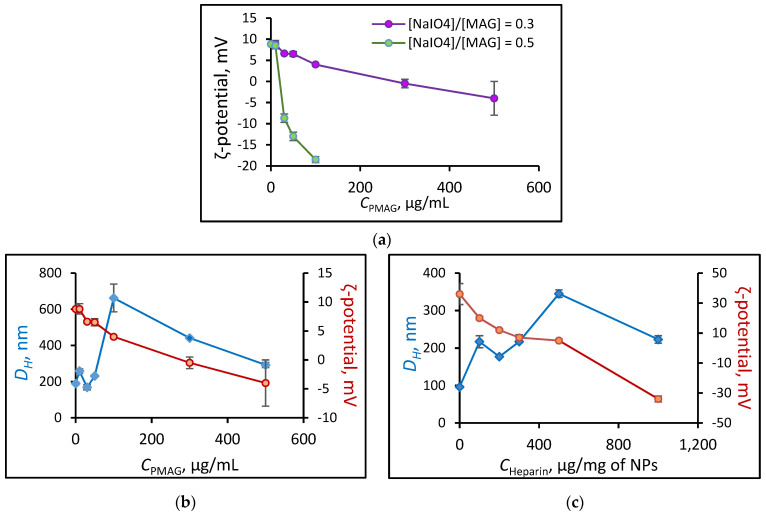
Dependence of (P(Lys-*co*-dl-Phe)-PMAG) particle ζ-potential on ox-PMAG concentration of different content of aldehyde groups (**a**) and dependence of P(Lys-*co*-dl-Phe) and P(Lys-*co*-d-Phe) particle size and ζ-potential on ox-PMAG (obtained at [NaIO_4_]/[MAG] = 0.3) (**b**) and heparin concentration, respectively (**c**) (0.01 M PBS, рН 7.4, C_NPs_ = 0.1 mg/mL).

Another approach to the preparation of polypeptide-glycopolymer NPs involves the formation of the polypeptide core from the self-assembled polymer, followed by its conjugation with oxidized glycopolymer (Figure 4). NPs based on P(Lys-*co*-dl-Phe) (*M_n_* = 26,200, *Ð* = 1.33 and [Lys]/[DL-Phe] = 1.1) were formed by self-assembly in water. The Schiff bases obtained due to the reaction of the amino groups of lysine and the aldehyde groups of the polymer were then reduced with sodium borohydride. The last reaction is an essential step not only because the formation of more stable single bond, but also reduction of unreacted aldehyde groups to hydroxyl when treated with sodium borohydride. The method for obtaining such particles (P(Lys-*co*-dl-Phe)-PMAG) was optimized in order to obtain systems with desired properties. Initially, the influence of the content of aldehyde groups in the glycopolymer on the ζ-potential of the formed particles was studied (Figure 5a). It was found that the ζ-potential of the particles decreases with an increase in the content of carbonyl groups in PMAG. This circumstance is obviously associated with a decrease in the content of amino groups and the appearance of hydroxyl groups on the particle surface. Furthermore, the physicochemical characteristics of the particles obtained at various polypeptide particle/glycopolymer ratios were investigated. The dependence of the particle hydrodynamic diameter (*D_H_*) with an increase in the initial glycopolymer/polypeptide particle ratio was non-linear (Figure 5b). With an increase in the concentration of PMAG the size increases in the initial section of the dependence at low concentrations of the glycopolymer, then decreases with an excess of glycopolymer. Apparently, at low concentrations of PMAG, the particles can crosslink due to an excess of amino groups, which leads to an increase in the particle size. In turn, the modification of the surface of the particles was accompanied with a reduction in the surface charge of NPs (Figure 5b).

A similar tendency in changing of the characteristics was also observed when NPs were electrostatically covered with heparin. Heparin as a strongly negative charged polymer interact with positive charged surface of particles due to electrostatic interaction. As in case of covalent covering of P(Lys-*co*-Phe) NPs with ox-PMAG, an increase in concentration of heparin led to a decrease in the ζ-potential and a nonlinear change in particle size. After reaching the maximum at a concentration 500 μg/mg of NPs, when a change in the sign of the ζ-potential occurred, the size decreased again, which is associated with an electrostatic repulsion of NPs covered with strongly negative heparin (Figure 5c).

It can be noted that depending on the discussed method of modification of particle surface, the point at which the particle surface charge is equal to 0 occurs at various concentrations of the coating polymer. Thus, it can be seen that at the isoelectric point, the particle size in both cases (PMAG and HEP coating) is about 350–450 nm (Figure 5).

### 3.3. Stability of Polymer Particles

**Figure 6:**
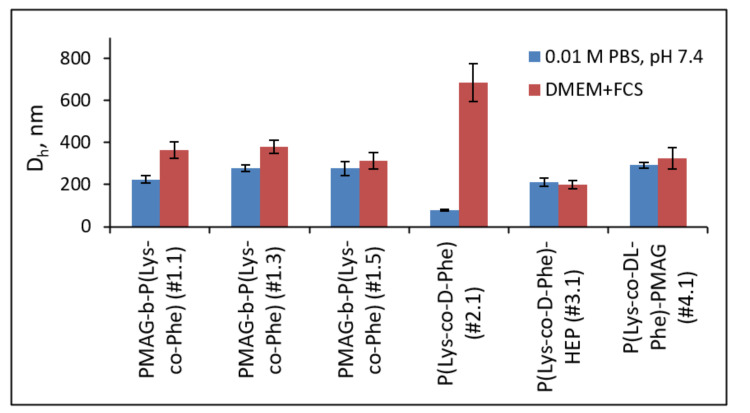
Hydrodynamic diameters of different nanoparticles (C = 0.1 mg/mL) in the buffer solution (0.01 M PBS, pH 7.4) and culture medium (DMEM + 10% FCS (*v*/*v*). The compositions of different polymer samples are provided in Table 2.

In current literature, the particle size is considered as one of the key parameters affecting their in vivo behavior. Moreover, the influence of particle charge on the stability of NPs in the bloodstream is discussed. However, often the study of particles’ stability is limited to their characterization at physiological pH in phosphate buffered saline (PBS). In this work, the stability of NPs has been studied in the presence of proteins, since their impact can strongly affect particle stability and size. As model media, PBS as well as cell culture medium (DMEM) containing fetal calf serum (FCS) were used. After the incubation of particles in the media for 24 h, their hydrodynamic size was measured by DLS (Figure 6).

Earlier we demonstrated that P(Lys-*co*-d-Phe)-based NPs aggregated in the DMEM + FCS culture medium, which can cause their cytotoxicity [35]. In turn, ones covered with heparin kept their size in protein-containing medium [35]. The results of this study confirm and complete the previously obtained results. The hydrodynamic diameter of anionic (P(Lys-*co*-d-Phe)-HEP) and neutral PMAG-*b*-P(Lys-*co*-Phe) (sample #1.5) and (P(Lys-*co*-dl-Phe)-PMAG) particles changed insignificantly and the particles remained stable to aggregation. On the contrary, the particles with a positive surface charge aggregated the more the higher ζ-potential they had. Thus, the stability of NPs in the presence of proteins is determined by their nonspecific interactions with FCS proteins. The aggregation of particles in the presence of serum proteins may be one of the factors that determine the cytotoxicity of the particles, which is discussed in the next section.

Besides the stability towards aggregation, there is a stability to biodegradation. In this case, the destruction of chemical bonds under the action of enzymes can cause a change in the particle structure. To study the enzymatic stability of the NPs, papain was used as an enzyme capable to cleave a peptide bond in a biodegradable polypeptide block. It was assumed that the introduction of D-phenylalnine instead of L-isomer as well as non-degradable glycopolymer into the polypeptide chain should improve the stability of NPs regarding their biodegradation. The biodegradation process in vitro was performed in 0.01 M PBS, pH 7.4, containing 0.5 mg/mL papain for 1 month and monitored as a function *D_H_* change.

The degradation of polymer particles based on L-α-amino acids was accompanied by an increase in their hydrodynamic diameter in a day after the incubation of the particles with the enzyme, and their sedimentation caused by strong aggregation was observed.

**Figure 7:**
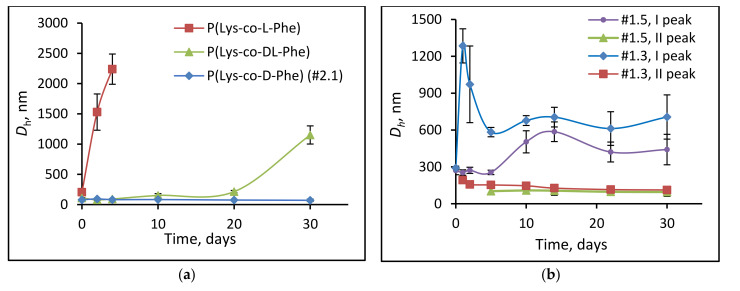
Changes in hydrodynamic diameters of NPs over time (DLS): (**a**) P(Lys-*co*-Phe) and (**b**) PMAG-*b*-P(Lys-*co*-Phe) NPs. Conditions of biodegradation: 0.01 M PBS, pH 7.4, T = 37 °C, *C*_NPs_ = 1.0 mg/mL; *C*_papain_ = 0.5 mg/mL. The compositions of different polymer samples are provided in Table 2.

About 15–20% decrease in size was observed for P(Lys-*co*-d-Phe) [35] (Figure 7). In this case, no particle aggregation was observed, and it seems that the cleavage of shorter P(Lys-*co*-d-Phe) occurs. In turn, the introduction of non-degradable PMAG in the copolymer and an increase of block length improved the degradation stability of NPs compared to ones based on random poly(l-amino acids). In particular, an increase in *D_H_* of PMAG-*b*-P(Lys-*co*-Phe) (sample #1.5, n_MAG_ = 80) based particles was observed after five days of incubation. At the same time, for this sample PMAG-*b*-P(Lys-*co*-Phe) another peak (about 100 nm) was appeared in DLS histograms. The observed peaks probably belong to the particles formed due to the cleavage of a degradable block and based on the random copolymer P(Lys-*co*-Phe). For the particles with a shorter PMAG block (sample #1.3, n_MAG_ = 22), the appearance of such a peak is observed already after 24 h of incubation with the enzyme, which is associated with less shielding of the internal degraded core from the action of the enzyme.

### 3.4. Cytotoxicity of Nanoparticles

In vitro cytotoxicity of polymer particles was studied using human embryonic kidney cells (HEK 293) and human lung carcinoma cells (A549) and CTB Assay after 24 and 72 h of incubation with NPs. The obtained results on cytotoxicity of the different tested NPs are summarized in Table 3. High cytotoxicity of P(Lys-*co*-d-Phe)-based NPs (IC_50_ < 51 μg/mL) probably can be associated with the aggregation of nanoparticles and the presence of big amounts of positively charged polylysine that can absorb with negatively charged surface of cells. After modification of these particles with heparin (P(Lys-*co*-d-Phe)-HEP) and, as a result, hiding surface amino groups and a positive charge, the cytotoxicity of NPs was significantly reduced (IC_50_ > 280 μg/mL, Table 3).

A similar effect was found for particles covered with PMAG shell (PMAG-*b*-P(Lys-*co*- Phe)-based particles and P(Lys-*co*-dl-Phe)-PMAG). In this case, the presence of a neutral charged glycopolymer on the surface of the particle partially compensated the negative effect of high positively charged PLys by reducing the interaction with serum proteins. As a result, for the sample #1.1 of PMAG-*b*-P(Lys-*co*-Phe), containing the largest number of lysine units, the cytotoxicity of the particles was reduced by at least two times compared to P(Lys-*co*-d-Phe)-based NPs (Table 3). At the same time, the sample #1.3 containing the smallest amount of lysine units did not show a toxic effect up to a concentration of 1 mg/mL. An increase in the length of neutral PMAG block, forming the hydrophilic shell of NPs, also led to a decrease in the toxic effect (sample #1.5 of PMAG-*b*-P(Lys-*co*-Phe)).

Thus, the particles based on random copolymers of lysine and phenylalanine, whose surface is modified by covalently bound synthetic glycopolymers or natural polysaccharides adsorbed on the surface, are characterized by much lower toxicity compared with unmodified analogues and have a great potential for use as drug delivery systems.

### 3.5. Cellular Uptake Study

**Figure 8:**
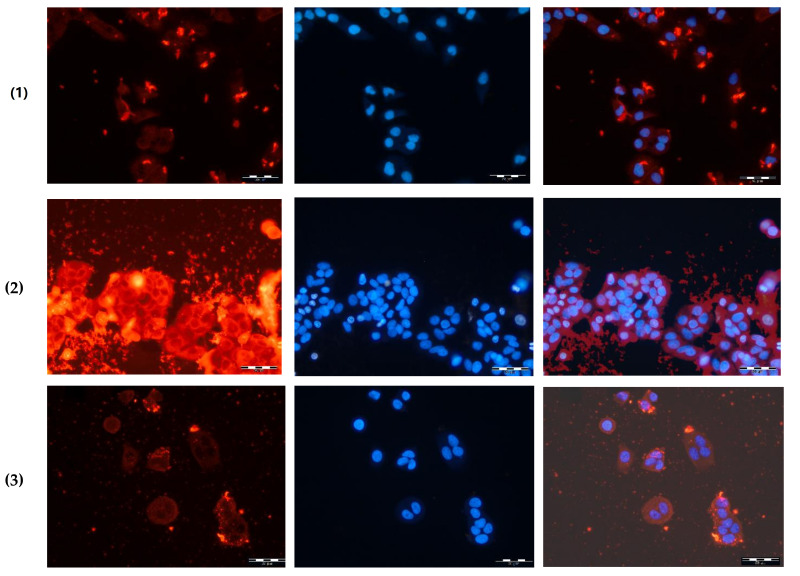
Fluorescent images of A549 cells treated with Cy_3_-labeled NPs: (**1**) PMAG-*b*-P(Lys-*co*-Phe), sample #1.5, (**2**) P(Lys-*co*-d-Phe), sample #2.1 and (**3**) (P(Lys-*co*-d-Phe)-HEP, sample #3.1 for 4 h (*C*_NPs_ = 60 µg/mL). Images from left to right show Cy_3_-labeled nanoparticles in cells (red), cell nuclei stained by DAPI (blue), and overlays of two images (×20). Scale bar: 50 μm.

To study the cellular uptake of nanomaterials, the fluorescent microscopy was applied. The PLys-based NPs were covalently labeled with Cy_3_ dye and incubated with A549 cells for 4 h. When the incubation time was over, the cell nuclei were stained with DAPI. The internalization of all kinds of nanoparticles indicated a relatively uniform distribution of Cy_3_ (red) in the cytoplasm around the nucleus (blue) (Figure 8). The coloration of cell cytoplasm allowed the conclusion that the nanoparticles were internalized into the cells. The highest fluorescence intensity was observed for cationic particles that is probably associated with the highest rate of their uptake.

For quantitative determination of particle cellular uptake, flow cytometry was applied. The kinetics of internalization was studied via incubation of Cy_3_-labeled particles with A549 cells and mouse macrophages (J774A.1 cell line). The cell fluorescence intensity (I_fl cell_) parameter is not informative if the particles themselves have different fluorescence intensities. Taking this into account, we used accumulation as parameter for comparison of different fluorescently labeled NPs. Accumulation was defined as the I_fl cell_ divided by the fluorescence intensity of the particles (I_fl NPs_). The values of accumulation were calculated as a percentage of the value obtained for the particles based on Cy_3_-labeled P(Lys-*co*-d-Phe) (sample #2.1) at C_NPs_ = 25 μg/mL after 24 h of incubation with A549 cells.

**Figure 9:**
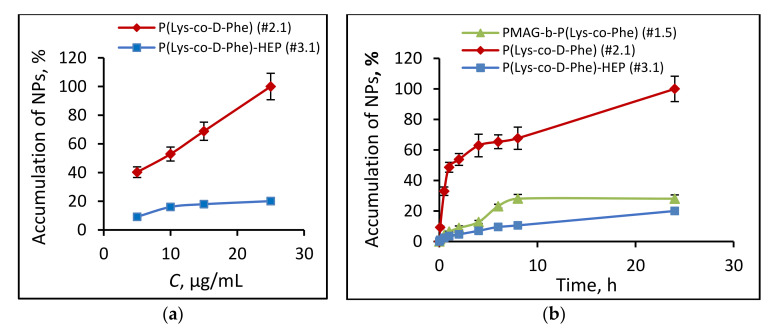
Dependence of NPs accumulation on their concentration (t = 24 h) (**a**) and kinetics of cell uptake of Cy_3_-labeled NPs (**b**): P(Lys-*co*-d-Phe) (sample #2.1), (P(Lys-*co*-d-Phe)-HEP (sample #3.1) and PMAG-*b*-P(Lys-*co*-Phe) (sample #1.5) NPs (*C*_NPs_ = 25 μg/mL, *C*_Cy3_ = 50 μg/mg of NPs, A549 cells). The compositions of different polymer samples are provided in Table 2.

As the first step, A549 cells were analyzed by regarding the accumulation of NPs in cells incubated with different concentrations of polymer nanoparticles. Different behavior of P(Lys-*co*-d-Phe) and (P(Lys-*co*-d-Phe)-HEP nanoparticles was observed. Thus, for positively charged NPs (P(Lys-*co*-d-Phe)), the accumulation almost linearly increased with increasing NPs concentration (Figure 9a), which may be attributed to the adsorption of positively charged particles on the cell membrane with their subsequent endocytosis. In turn, for (P(Lys-*co*-d-Phe)- HEP NPs at a concentration of >10 μg/mL, an increase in the concentration of particles in the culture medium did not lead to an increase of accumulation, probably due to the worse interaction of negatively charged surface of cell surface with the like charged NPs.

The accumulation for (P(Lys-*co*-d-Phe)-based NPs was considerably increased at initial hours that can be associated with the adsorption of positively charged particles on the cell membrane. At the same time, the modification of nanoparticles with heparin, in which there is a change in surface charge of NPs from positive to negative, affects not only the toxicity of polymer particles, but also the profile of their accumulation in cells. Thus, the particles based on (P(Lys-*co*-d-Phe)-HEP had an accumulation rate 10 (at the initial stages, t < 4 h) and five (t = 24 h) times lower compared with unmodified analogue (Figure 9b). The accumulation of neutral charged (PMAG-*b*-P(Lys-*co*-Phe)-based NPs increased rapidly at initial hours and reached the maximum after 8 h of incubation (Figure 9b).

**Figure 10:**
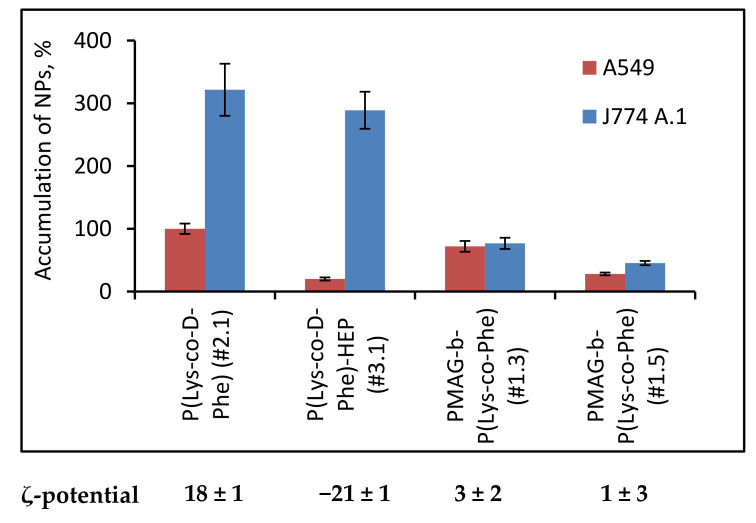
Dependence of NPs accumulation in A549 cells and J774 A.1 macrophages on their composition and surface properties (*C*_NPs_ = 25 μg/mL, *C*_Cy3_ = 50 μg/mg of NPs, t = 24 h).

It is known that clearance rate of NPs is dependent on their surface properties where the interactions with the reticuloendothelial system tend to increase with charge. Negative surface charge can either increase, decrease, or have no impact on the blood clearance of nanoparticles, but a positive charge generally has a negative effect upon exposure to plasma [58]. To evaluate the effect of surface charge on the uptake by macrophages, NPs with different surface charge, namely strong positive P(Lys-*co*-d-Phe), negative P(Lys-*co*-d-Phe)-HEP, slightly positive (sample #1.3) and neutral (sample #1.5) of PMAG-*b*-P(Lys-*co*-Phe) were incubated with J774A.1 macrophages (Figure 10).

Despite that NPs with negative and positive surface charge had a different accumulation in cancer cells, they had a similar uptake by macrophages. Introduction of a neutral block of PMAG, in turn, strongly decreased accumulation in J774A.1 cells compared to cationic and anionic analogues. The latter circumstance will favor an increase of the circulation time in the bloodstream. In addition, an increase in the length of the PMAG block led to a decrease in the uptake of PMAG-*b*-P(Lys-*co*-Phe) NPs by macrophages.

Finally, we compared the uptake of developed NPs with widely used biodegradable poly(lactic acid)-based (PLA) nanospheres. Introduction of PEG block onto NPs’ surface is a common technique for creation of systems with “stealth” properties [59]. For this reason, PLA (*D_H_* = 84 ± 1 nm, PDI = 0.13 ± 0.05) and PEG-*b*-PLA (*D_H_* = 90 ± 10 nm, PDI = 0.24 ± 0.01) NPs were used for comparison. For particle modification, different approaches, including the activation of carboxylic groups of PLA NPs with N-hydroxysuccinimide to form activated NHS esters as well as oxidation of glucopyranose ring of PMAG with NaIO_4_ to form reactive aldehyde groups, were applied. Then, NPs were covalently labeled with Cy_5_-NH_2_ dye using the reaction of aldehydes or activated carboxylic groups with amino group of dye.

**Figure 11:**
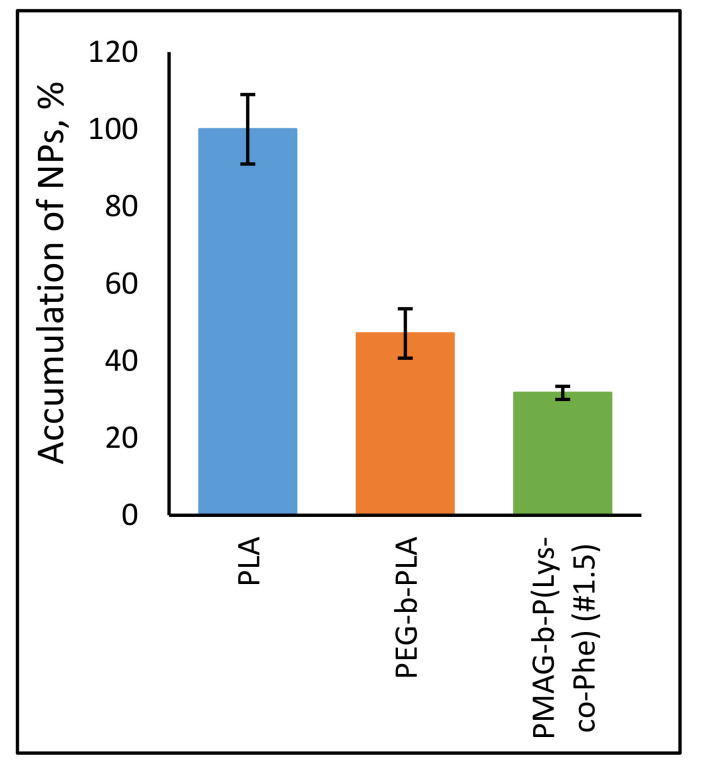
Uptake of the Cy_5_-labeled NPs by J774 A.1 macrophages. (*C*_NPs_ = 50 μg/mL, *C*_Cy5_ = 3 μg/mg of NPs, t = 6 h).

The accumulation of PLA NPs was maximal, indicating the strong macrophage uptake (Figure 11). This value was set as 100% for this experiment. At the same time, the introduction of PEG block into the structure of the polymer particles reduced the macrophage uptake about two times, which is in a good agreement with literature data. The reduced uptake was observed for developed ternary copolymers in comparison with control PLA and even PEG-*b*-PLA NPs. As for the previous case, it was confirmed that the presence of a neutral hydrophilic shell, formed by the PMAG-block, reduces the uptake of particles and such NPs can be considered as nanoobjects with reduced macrophage uptake.

### 3.6. Development of Paclitaxel Delivery Systems

Paclitaxel (PTX) is an effective antitumor drug against a wide variety of tumors, including ovarian, metastatic breast, non-small cell lung cancers, and so on [60]. However, the clinical applications of PTX have been hampered by its low aqueous solubility and bioavailability [61]. The currently available formulation of PTX has been reported to cause serious side effects, such as hypersensitivity reactions, nephrotoxicity, neurotoxicity, and cardiotoxicity [62,63]. With the aim to increase solubility and bioavailability of paclitaxel, as well as enhance drug safety, prolong the blood circulation time, improve its efficacy, and reduce side effects, the new formulations of paclitaxel are being developed. The current literature discusses various paclitaxel delivery systems, including liposomes [64], albumin-bound-paclitaxel nanoparticles [65], solid lipid nanoparticles [62], micelles [66], dendrimers [67], films [68], polymersomes [69], and hydrogels [70].

**Figure 12:**
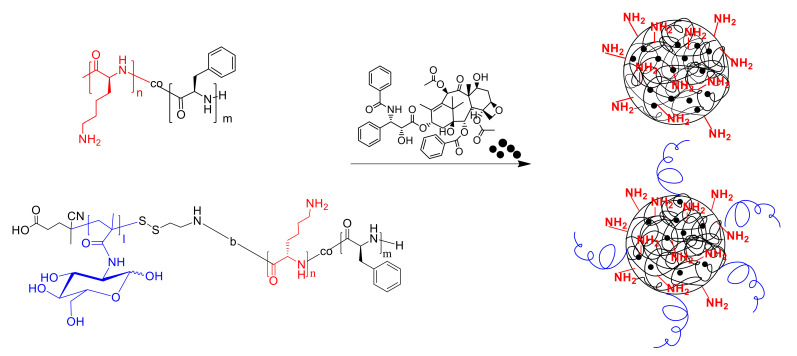
Schematic representation of paclitaxel-loaded NPs formulations.

In this research, the encapsulation of the hydrophobic drug paclitaxel (Figure 12) into NPs was carried out as previously described [54]. Briefly, the mixture of copolymer and drug dissolved in DMSO was lyophilized to achieve high encapsulation efficiency and minimize drug loss.

The characteristics of obtained paclitaxel formulations are summarized in Table 4. As seen, the encapsulation of the hydrophobic drug paclitaxel into polymer particles increased with increasing hydrophobicity of the polymer. In addition, a tendency to increase in size and a slight change in the ζ-potential of the particles was observed, when the drug was encapsulated into the NPs (Table 4).

In vitro antitumor activity of paclitaxel-loaded particles on A549, and MCF-7 cancer cells was evaluated (Table 4) compared to commercially available Paclitaxel formulation (PTX-LANS), which is a solution of paclitaxel, using excipients macrogol glyceryl hydroxystearate and ethanol.

It can be seen that the modification of cationic particles with heparin increases IC_50_ values, which can be attributed to a decrease in the rate of penetration of particles into cells and, probably, the rate of release of the drug from the polymer matrix. Ternary copolymers PMAG-*b*-P(Lys-*co*-Phe) have proven to be the most promising candidates for further study as delivery systems. Paclitaxel delivery systems based on these hybrid copolymers have high activity against cancer cells, while the polymer particles themselves were not cytotoxic on various human cells. In addition, compared to their cationic and anionic counterparts, they have a low uptake by macrophages, which will increase their circulation time in the bloodstream. Moreover, in comparison to previously synthesized di-block copolymers PMAG-*b*-poly(amino acid) [54], they contain reactive amino groups, which are capable of both electrostatic and covalent binding of various substances (drugs, vectors). The disulfide bond between PMAG and P(Lys-*co*-Phe) blocks could provide the sensitivity of such systems to glutathione-mediated disruption of the NPs inside a cell providing targeted release.

The use of encapsulated forms of the drug allowed achievement comparable efficacy and, in some cases, increasing of biological activity of a drug compared to commercially available Paclitaxel-LANS. At the same time, such formulations significantly increase the solubility of paclitaxel and may increase its bioavailability, prolong the circulation half-life, reduce side effects, and, as a consequence, improve drug efficacy.

## 4. Conclusions

In this study, two novel different approaches have been applied for preparation of polypeptide-glycopolymer nanoparticles. The first one includes the synthesis of block-random copolymers consisting of polypeptide and glycopolymer and capable of self-assembly into polymer particles. Namely, the novel block-random copolymers, consisting of amphiphilic biodegradable random polypeptide block and hydrophilic synthetic glycopolymer poly(2-deoxy-2-methacrylamido-d-glucose) (PMAG) have been synthesized and carefully characterized. Another approach includes the post-modification of preformed polypeptide polylysine-based particles with the functionalized glycopolymer.

It was shown that structure and composition of initial copolymers affected on their physicochemical properties (size, ζ-potential, PDI). Positive particles’ charge led to their aggregation in model physiological medium, that can be adjusted by surface modification with neutral or negatively charged polysaccharides. Using the fluorescent-labeled nanoparticles, it was shown that the developed nanoparticles have penetrated inside the cells via non-specific cellular uptake.

It also has been found that cationic NPs based on random copolymers of amino acids whose surface is modified in various ways (covalently bound synthetic glycopolymers/natural polysaccharides adsorbed on the surface) are characterized by a lower rate of penetration into cancer cells and much lower toxicity compared to unmodified analogues. NPs with negative and positive surface charge had a different accumulation in cancer cells and similar fast uptake by macrophages. The introduction of a neutral block of PMAG strongly decreased the cytotoxicity and uptake by macrophages compared to cationic and anionic analogues. These features make them very attractive for further development of targeted drug delivery formulations.

## Figures and Tables

**Table 1:** The characteristics of synthesized amphiphilic PMAG_l_-*b*-P(Lys_n_-*co*-Phe_m_).

Sample #	[*M*_1_ + *M*_2_] */ [PMAG]	[*M*_1_]/ [*M*_2_]	SEC **		HPLC	^1^H NMR	
			*M_n_*	*Ð*	[Lys]/ [Phe]	[MAG]/[Lys(Z)]/[Phe]	*l*_MAG_:*n*_Lys_: *m*_Phe_
1.1	75	2/1	28,600	1.49	1:0.4	0.4:1:0.5	22:55:27
1.2	75	1/1	30,700	1.31	1:0.9	0.8:1:0.8	22:31:25
1.3	75	1/2	25,900	1.38	1:1.6	1.1:1:1.5	22:20:30
1.4	150	1/1	29,500	1.44	1:0.9	0.4:1:0.9	22:55:50
1.5	75	1/1	34,500	1.42	1:0.9	2.8:1:0.8	80:29:23

* *M*_1_ = NCA Lys(Z), *M*_2_ = NCA Phe; ** Determined for Z-protected samples.

**Table 2:** Composition of P(Lys_n_-*co*-Phe_m_) and PMAG_l_-*b*-P(Lys_n_-*co*-Phe_m_) copolymers and characteristics of self-assembled nanoparticles in 0.01 M PBS, pH 7.4.

Sample	*#*	*l*_MAG_	*n*_Lys_	*m*_Phe_	*l*_MAG_:*n*_Lys_: *m*_Phe_	*D_H_*	*PDI*	ζ-potential
PMAG-*b*- P(Lys-*co*-Phe)	1.1	22	55	27	0.4:1:0.5	230 ± 20	0.32 ± 0.08	8 ± 1
	1.2	22	31	25	0.8:1:0.8	170 ± 10	0.22 ± 0.03	5 ± 2
	1.3	22	20	30	1.1:1:1.5	290 ± 20	0.36 ± 0.04	2 ± 1
	1.4	22	55	50	0.4:1:0.9	180 ± 10	0.31 ± 0.05	6 ± 1
	1.5	80	29	23	2.8:1:0.8	270 ± 30	0.29 ± 0.04	1.5 ± 0.1
P(Lys-*co*-d-Phe)	2.1	-	34	34	0:1:1	71 ± 3	0.22 ± 0.05	36 ± 6
	2.2	-	72	17	0:1:0.23	96 ± 3	0.22 ± 0.01	36 ± 7
	2.3	-	87	9	0:1:0.1	150 ± 10	0.23 ± 0.01	37 ± 1
P(Lys-*co*-d-Phe)-HEP *	3.1	-	34	34	0:1:1	210 ± 20	0.23 ± 0.02	−38 ± 2
	3.2	-	72	17	0:1:0.23	210 ± 20	0.23 ± 0.02	−38 ± 2
	3.3	-	87	9	0:1:0.1	220 ± 20	0.20 ± 0.01	−34 ± 1
P(Lys-*co*-dl-Phe)-PMAG **	4.1	-	35	32	-:1:0.9	290 ± 10	0.36 ± 0.06	−4 ± 4

* P(Lys-*co*-d-Phe)-HEP particles were obtained at concentration of HEP equal to 1000 µg/mg of polypeptide particles; ** P(Lys-*co*-dl-Phe)-MAG particles were obtained at concentration of ox-MAG ([NAIO_4_]/[MAG]=0.3) equal to 5000 µg/mg of polypeptide particles.

**Table 3:** Cytotoxic effect of developed nanoparticles.

Polymer NPs	Polymer Sample *	Time, h	Cell Line	IC_50_, μg/mL
PMAG-*b*-P(Lys-*co*-Phe)	1.1	24	HEK293	130 ± 10
			BEAS-2B	37 ± 2
		72	HEK293	130 ± 20
			BEAS-2B	38 ± 1
	1.3	24	HEK293	>1000
			BEAS-2B	>1000
		72	HEK293	>1000
			BEAS-2B	>1000
	1.5	24	HEK293	>1000
			BEAS-2B	>1000
		72	HEK293	>1000
			BEAS-2B	>1000
P(Lys-*co*-d-Phe)	2.1	24	HEK293	28 ± 3
			BEAS-2B	9.6 ± 0.3
		72	HEK293	25 ± 1
			BEAS-2B	8.3 ± 0.4
	2.3	24	HEK293	45 ± 3
			BEAS-2B	7.1 ± 0.1
		72	HEK293	50 ± 2
			BEAS-2B	6 ± 0.1
P(Lys-*co*-d-Phe)-HEP	3.1	24	HEK293	>1000
			BEAS-2B	>1000
		72	HEK293	280 ± 30
			BEAS-2B	>1000
P(Lys-*co*-dl-Phe)-PMAG	4.1	24	HEK293	>1000
			BEAS-2B	>1000
		72	HEK293	>1000
			BEAS-2B	>1000

* The polymer compositions of samples are given in Table 2.

**Table 4:** Characteristics of paclitaxel-loaded NPs and cytotoxic effect of the developed paclitaxel formulations.

Formulation	Polymer Sample *	EE (%)	LC (μg/mg of NPs)	Characteristics of PTX-Loaded NPs		IC_50_, ng/mL	
				*D_H_* (PDI)	ζ-potential (mV)	A549	MCF-7
PTX-LANS	−	−	−	−	−	2.0 ± 0.3	4 ± 1
PMAG-*b*-P(Lys-*co*-Phe)	1.3	95	95	430 (0.25)	−3.2 ± 0.4	4.4 ± 0.6	4.1 ± 0.5
	1.5	87	87	410 (0.27)	2.0 ± 0.4	2.7 ± 0.3	6.9 ± 0.6
P(Lys-*co*-d-Phe)	2.1	95	95	250 (0.14)	32 ± 1	1.8 ± 0.3	15 ± 1
	2.2	93	93	160 (0.21)	27 ± 3	4.8 ± 0.5	4.5 ± 0.5
	2.3	89	89	210 (0.18)	30 ± 2	7.0 ± 0.8	2.8 ± 0.3
P(Lys-*co*-d-Phe)-HEP	3.1	95	95	230 (0.22)	−34 ± 2	13 ± 1	24 ± 2
	3.2	93	93	230 (0.22)	−30 ± 2	7.9 ± 0.7	−
	3.3	89	89	250 (0.19)	−30 ± 2	22 ± 3	−

* The polymer compositions of samples are given in Table 2.

## Data Availability

Data available within the article or its Supplementary Materials.

## Supplementary Materials

The following supporting information can be downloaded at: https://www.mdpi.com/article/10.3390/polym14091677/s1, Figure S1: Transmission electron microscopy (TEM) images of P(Lys-*co*-d-Phe) (sample #2.2) (a) and PMAG-*b*-P(Lys-co-Phe) (sample #1.5) (b).
