# Supplementary material for: Biocompatible Nanoparticles Based on Amphiphilic Random Polypeptides and Glycopolymers as Drug Delivery Systems
Source: Polymers (Basel). 2022 Apr 20;14(9):1677. doi: 10.3390/polym14091677 (PMC9104652; doi:10.3390/polym14091677)
Supplement: Supplementary file 1 [file polymers-14-01677-s001.zip › polymers-1666076-supplementary.pdf]

polymers

MDPI

Supplementary Materials

Biocompatible Nanoparticles Based on Amphiphilic Random Polypeptides and Glycopolymers as Drug Delivery Systems

Natalia Zashikhina [1], Mariia Levit [1], Anatoliy Dobrodumov [1], Sergey Gladnev [2], Antonina Lavrentieva [3], Tatiana Tennikova [2] and Evgenia Korzhikova-Vlakh [1,*]

[1] Institute of Macromolecular Compounds, Russian Academy of Sciences, Bolshoy pr. 31, 199004 St. Petersburg, Russia; nzashihina@bk.ru (N.Z.), musia_1@yahoo.com (M.L.); anatoliy.dob@gmail.com (A.D.);

[2] Institute of Chemistry, Saint-Petersburg State University, Universitesky pr. 26, 198504 St. Petersburg, Russia; V.); st069020@student.spbu.ru (S.G.); tennikova@mail.ru (T.T.);

[3] Institute of Technical Chemistry, Gottfried-Wilhelm-Leibniz University of Hannover, 30167 Hannover, Germany; lavrentieva@iftc.uni-hannover.de (A.L.);

* Correspondence: vlakh@hq.macro.ru.

(a) **(b)**

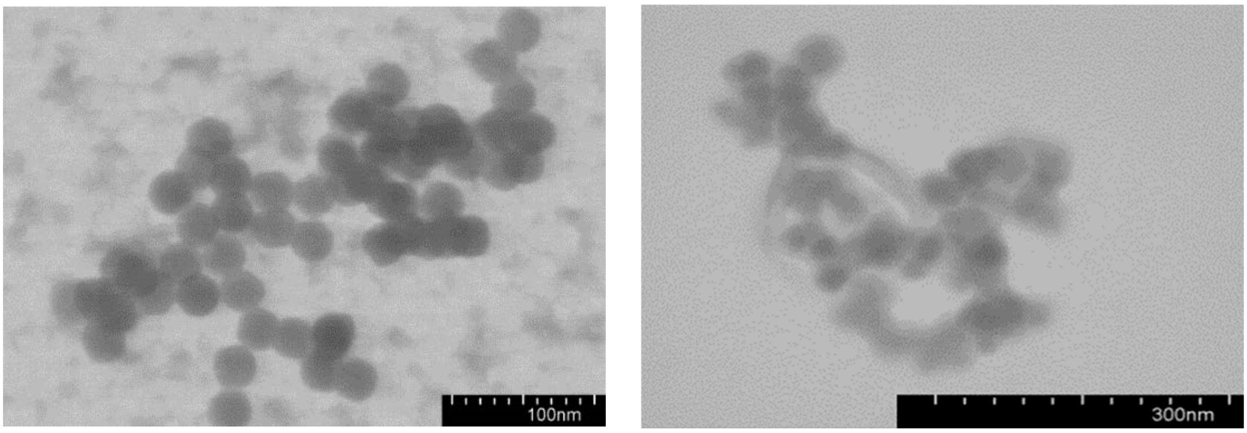

Figure S1. Transmission electron microscopy (TEM) images of P(Lys-co-D-Phe) (sample #2.2) (**a**) and PMAG-b-P(Lys-co-Phe) (sample #1.5) (**b**).
